# Supplementary figures and images for: Leishmania highjack host lipid body for its proliferation in macrophages by overexpressing host Rab18 and TRAPPC9 by downregulating miR-1914-3p expression
Source: PLoS Pathog. 2024 Feb 27;20(2):e1012024. doi: 10.1371/journal.ppat.1012024 (PMC10898768; doi:10.1371/journal.ppat.1012024)

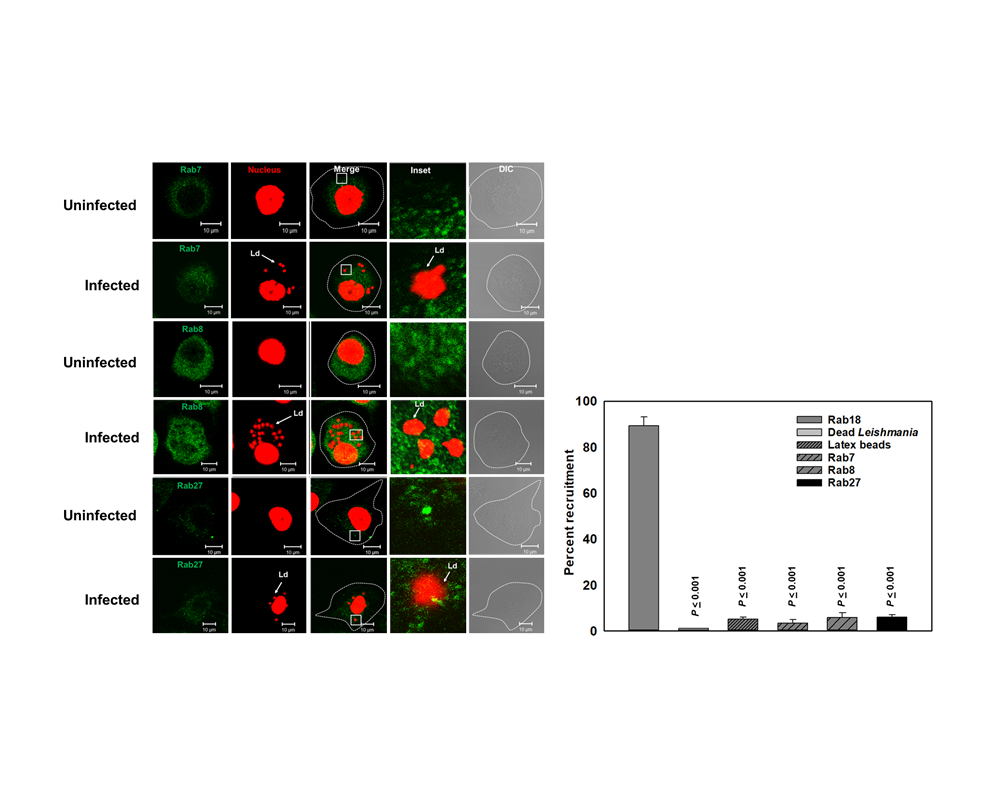

Supplement: S1 Fig — (TIF) [file ppat.1012024.s001.tif]

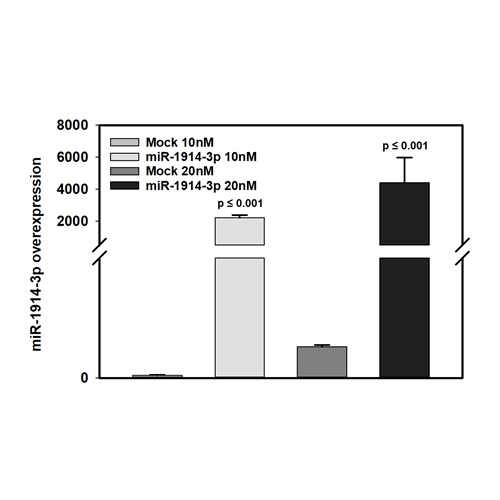

Supplement: S2 Fig — (TIF) [file ppat.1012024.s002.tif]

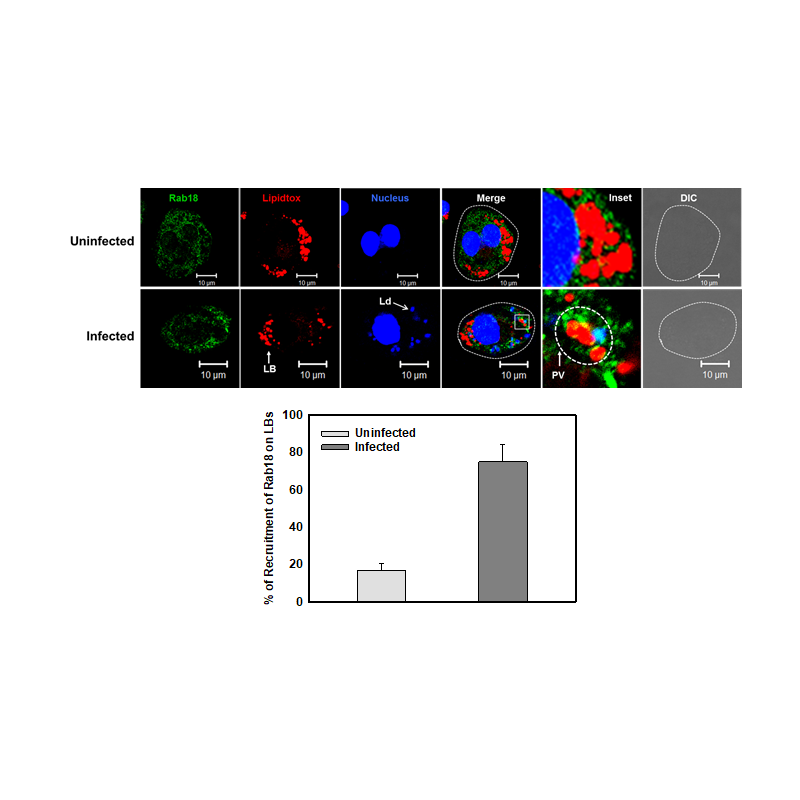

Supplement: S3 Fig — (TIF) [file ppat.1012024.s003.tif]

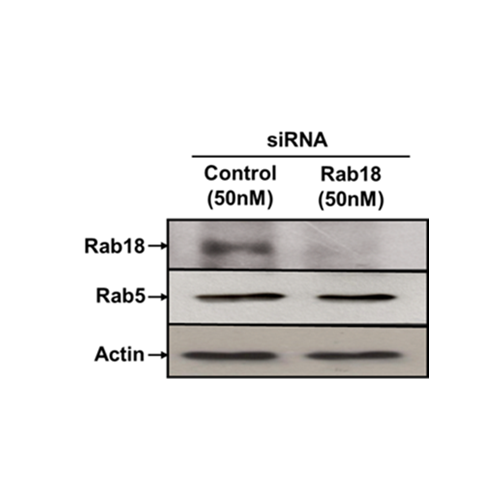

Supplement: S4 Fig — (TIF) [file ppat.1012024.s004.tif]

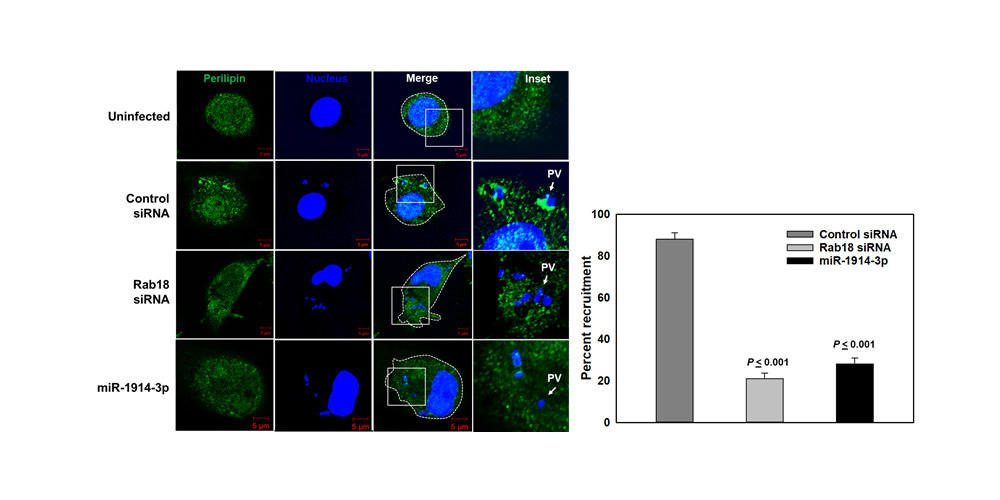

Supplement: S5 Fig — (TIF) [file ppat.1012024.s005.tif]
