# Supplementary material for: Leishmania highjack host lipid body for its proliferation in macrophages by overexpressing host Rab18 and TRAPPC9 by downregulating miR-1914-3p expression
Source: PLoS Pathog. 2024 Feb 27;20(2):e1012024. doi: 10.1371/journal.ppat.1012024 (PMC10898768; doi:10.1371/journal.ppat.1012024)
Supplement: Attachment — Submitted filename: Point by Point response.pdf [file ppat.1012024.s006.pdf]

Point by Point response:

Reviewer 1:

Comment: The manuscript by Sood et al. "Leishmania highjack host lipid body to acquire fatty acids by overexpressing Rab18 and TRAPPC9 by downregulating miR-1914-3p expression in macrophages" sets out to dissect the mechanism of fatty acid uptake from the host by the kinetoplastid parasite Leishmania donovani. Since Leishmania is a major human pathogen and serves as a valuable cell biological model understanding the uptake mechanism of fatty acids is significant from an intervention strategy and from a general knowledge perspective.
Through a set of experiments (microscopy, western blot, semiquantitative PCR) the authors conclude that dicer is degraded by gp63, which reduces the expression of miR-1914-3p. This in turn upregulates Rab18 and TRAPPC9, which induces recruitment of fatty acid containing lipid bodies to the parasitophorous vacuole. This set of events would provide a significant, comprehensive insight into the mechanism of fatty acid uptake by Leishmania donovani. However, the manuscript lacks scientific rigour and suffers from over-interpretation (see major issues).

Response: We appreciate the reviewer for nicely summarizing our results and stating that our finding provide a significant, comprehensive insight into the mechanism of fatty acid uptake by *Leishmania donovani*. However, he has commented that manuscript suffers from over-interpretation which we have appropriately corrected in the revised manuscript.

Major issues:

The manuscript relies mainly on two lines of evidence: the up- or down-regulation of molecules and the localisation of molecules depending on infection status. Most experiments are not explained in sufficient details and lack appropriate controls.

Question 1: Some of the bands in the western blots (especially loading controls) are already in saturation introducing artefacts and making quantification unreliable. This increase in expression is also not necessarily supported by the provided microscopy pictures.

Reply 1: We have provided appropriate blots in the revised manuscript as suggested by the reviewer. However, I would like to state that over expression and recruitment of Rab18 and TRAPPC9 by PV in infected macrophages are well documented by confocal microscopy (Fig.1d, Fig.1f, Fig. 2c) and qPCR (Fig. 1b. Fig.2b) which is also appreciated by other reviewers. We have mentioned all loading control in the figure legend of the revised manuscript.

Question 2: PFA potentially destroys surface structures and hence the conclusion that live *Leishmania* are necessary cannot be drawn from this experiment.

Reply 2: We have used PFA fixed (dead) parasites and latex beads as controls and shown that dead parasites and latex beads containing phagosomes do not recruit Rab18 and lipid bodies. We have used PFA fixed (dead) parasites as a control of similar size particles like live *Leishmania* as phagocytic efficacy and trafficking may vary depending on particle size [*Infect. Immun.* (1996) 64, 3877–3883]. Moreover, we and others have used PFA fixed, glutaraldehyde fixed or heat killed pathogens as control in understanding trafficking of

intracellular pathogens in host cells in several studies [*J. Biol. Chem.* (2000) 275, 16281-16288; *J. Cell Biol.* (2000) 148, 741-753; *PLoS Pathog.* (2017) 13, e1006459]. Thus, PFA fixed parasites is just like a particle which will not be able to secrete any factor from parasites as PFA potentially destroys surface structures as said by the reviewer. Therefore, I believe reviewer will agree that PFA fixed dead parasites act like similar-sized particles. Thus, our results demonstrated that live *Leishmania* is required for the recruitment of Rab18 and lipid bodies from host cells.

Question 3: Microscopy data:

Question I. No correlation between the structures detected by Rab18 and the LipidTOX stain is shown. No data on the acquisition is provided (how many cells?, settings?, were the same setting used?...).

Reply I. I would like to state that colocalization of Rab18 and the LipidTOX is clearly visible (yellow) in the Fig. 4f of the reviewed manuscript. I would like to submit that there is technical limitation of labelling PV by Lamp1 in this image as we have already used three colours in the image. Therefore, we had to mark PV by dotted lines based on the fact that vesicular membrane of Ld-PV is not closely associated with amastigotes and size of the PV is about 10µm [*J. Parasitol.* (2006) 92, 1162-1170]. All the images were captured using similar settings in the confocal microscope. At least 100 PVs were analysed to determine the recruitment of Rab18 and LipidTox stained Ld-PVs and provided the quantification data in the revised manuscript. Representative images were provided in the manuscript as stated in the figure legend.

Question II. Scale bars are only just visible when one zooms in. PV is never shown (DIC image or specific marker), but drawn onto the figure – this is an interpretation by the authors and not primary data that can be evaluated.

Reply II. We have provided the staining of PV by LAMP1 in the revised manuscript as suggested by the reviewer. We have appropriately drawn the scale bar and included DIC images in the revised manuscript.

Question III. Given the very weak signal of Trappc9 in uninfected cells, it would be good to show specificity and controls using only secondary antibodies.

Reply III. I would like to submit that the expression of TRAPPC9 is clearly visible in uninfected cells and its overexpression in *Leishmania*-infected cells as shown in Fig. 2c of the reviewed manuscript. Thus, the staining of TRAPPC9 is specific. We have done the new experiment and included a better image in the revised manuscript.

Question IV. Fig. 4A - no nuclei visible in uninfected host cell, raises question of consistency of data/setting/robustness, again quantification murky, not enough detail provided to interpret the data, recruitment or non-recruitment of LB is not obvious

Reply IV. We have included new experiment in the revised manuscript taking care of the points suggested by the reviewer.

Question V. Fig. 4D –5B "lipid markers nearby PV" – what does "nearby mean"? What were the objective criteria for this analysis?

Reply V. I have already explained that nearby means on/within the PV containing *Leishmania* in infected cells which are marked by dotted lines in the reviewed manuscript. I would like to state that this is clearly shown in LAMP1 stained PV in the Fig.5b in reviewed manuscript. However, we have provided LAMP1 staining on PV in Fig.1d, 1c, 2c, 4a, 4b and 4c and have shown that respective markers are localized on/within the PV in the revised manuscript.

Fig.1d.

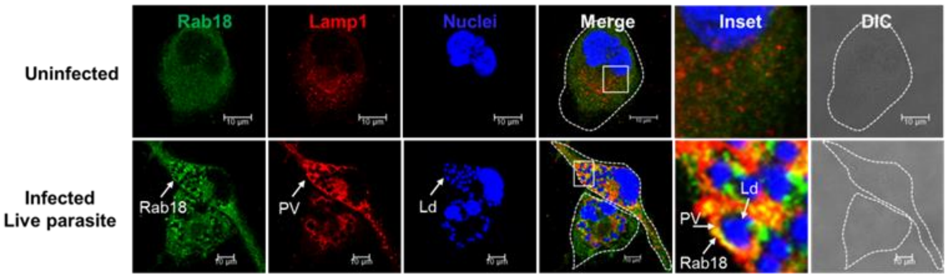

Fig.2c.

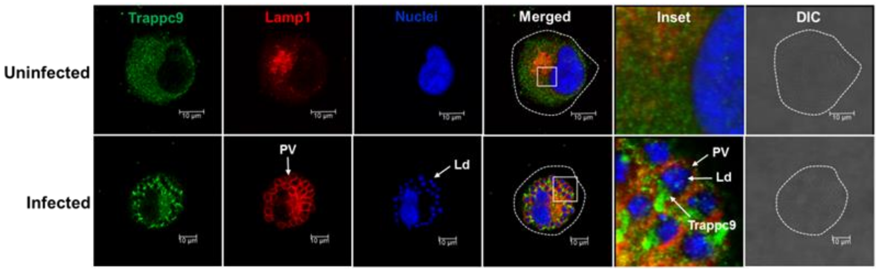

Fig.4a.

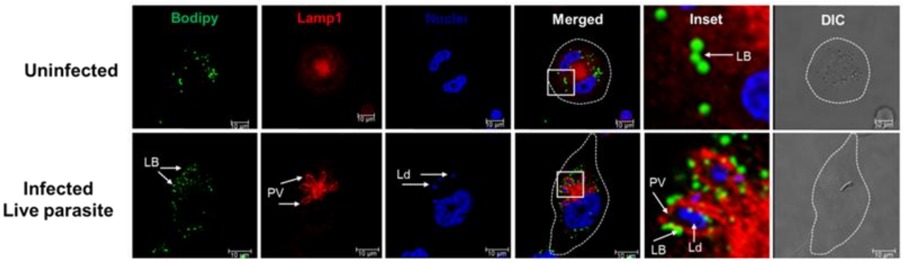

Fig.4b.

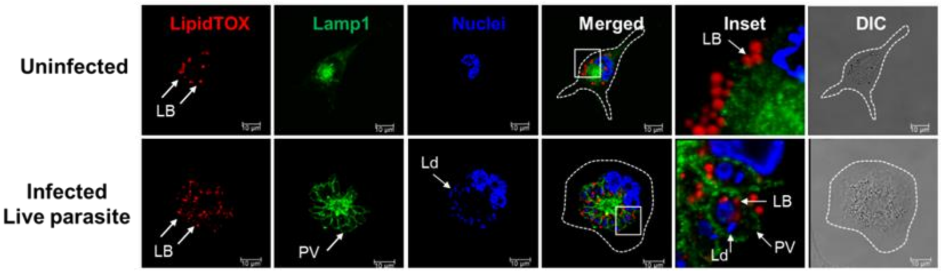

Fig.4c.

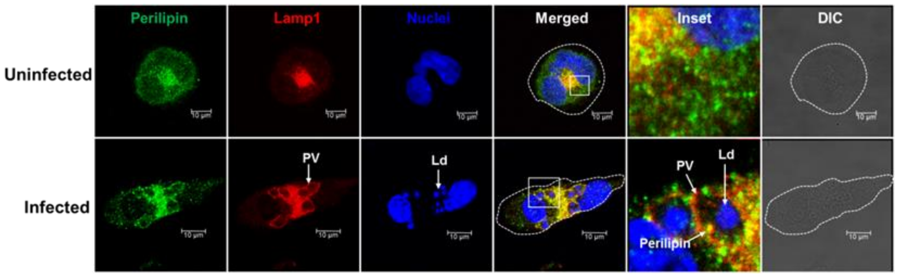

Question VI. Fig. 4E - How where the number of lipid bodies determined (Only one section or were z-stacks produced?) According to the text, 50 macrophages were analysed (per condition?), but the figure only shows 5 data points.

Reply VI. I would like to submit that number of lipid bodies and size were determined using a software which count number of pixels of a flurochrome in the cells and show approximate size of each dot. This is well established method [Cell Metab. (2018) 27:886-97]. I would like to state that we have analysed 50 macrophages as mentioned in the text, most of the dots are within the bar and only larger deviations are outside the bar in the reviewed manuscript which might have confused the reviewer. We have included a different plot in the revised manuscript.

Question VII. Fig. 4G - “In addition, we found that calnexin-labelled ER membrane is also recruited on PVs indicating plausible mechanism how newly synthesized LBs bearing ER membrane are recruited on PVs (Fig.4g).” This is a throw-away comment – no analysis of this data is provided nor is this explored in any detail.

Reply VII. In the Fig.4g, we have shown that Ld-PV also recruit Calnexin, an ER-membrane marker along with lipid bodies. Previous studies have also shown that LD-PV recruits calnexin and thereby induces the fusion of ER membrane with Ld-PV by SNARE protein Sec22b [Cell Microbiol. (2010) 12:1480-94]. As lipid bodies bud from the ER, bearing ER membrane (Calnexin), therefore, the recruitment of lipid bodies and calnexin in/on Ld-PV indicate a plausible mechanism how newly synthesized LBs bearing ER membrane are recruited on PVs. As fusion of ER membrane with Ld-PV have already been demonstrated, thus, our statement that LBs bearing ER membrane recruited on PVs is based on our results and previous studies. Therefore, we have written ‘indicating plausible mechanism’ not saying ‘our result demonstrated’. We have done new experiment and shown that calnexin is recruited on Ld-PV similar to Lamp1 staining on PV in the revised manuscript.

Question VIII: In general, Figure legends and Material and Methods lack important information and do not allow for the evaluation of the data nor for its reproducibility (e.g. Fig. 3f – incubation time for gp63).

Reply VIII. I would like to submit that some of the methodologies are in brief as we have reported in our previous publications along with reference. However, we have included appropriate figure legends and detailed methodology in the revised manuscript.

Question IX: It is hard to follow the logic of the study: often it is not obvious, why a particular experiment was undertaken as a next step. This becomes clearer in the discussion,

where the authors outline their chain of argument, however, they often use circumstantial rather than actual evidence (e.g. by citing situations from unrelated organisms).

The manuscript would benefit from a more cautious interpretation of the data (expecially given the experimental shortcomings listed above). The discussion is spiked with statements as "To unequivocally prove..", "we have clearly demonstrated...", "we have precisely addressed how..." or "Toxoplasma is the only other intracellular parasite..." (Have ALL other intracellular parasites been investigated?).

Often there is a functional link implied, where there is no evidence provided: e.g. "The enhance recruitment of Rab18 and its GEF on PV facilitates the docking of LB on PV and thereby parasites acquire fatty acids from LBs for their survival." (no acquisition of fatty acids was shown).

"Our results also indicate that overexpressions of Rab18 and TRAPPC9 induce the biogenesis of LBs in infected cells as biogenesis of LBs in cells are shown to be predominantly regulated by Rab18 and its GEF [10, 11]." (Rab18 and GEF have been identified in these publications as important (but not predominant) factors and biogenesis of LBs has not been shown in the submitted study.)

Reply IX: We appreciate reviewer as he has found that the link of our results are clearer in the discussion. We have rewritten our text withdrawing the statement like "unequivocally prove"; "clearly demonstrated"; "precisely addressed" and use the word like suggested, indicated etc. in the revised manuscript as suggested by the reviewer.

Reviewer has stated that "no acquisition of fatty acids was shown". In contrast, he has said in his comment that "this set of events would provide a significant, comprehensive insight into the mechanism of fatty acid uptake by *Leishmania donovani*". I am sorry to inform that the reviewer possibly misinterpreted the Lipid bodies staining methods by different dyes. We have used BODIPY™ 493/503 and LipidTOX™ to stain the LBs and anti-perilipin antibody is used to detect lipid droplet-associated protein. Thus, all these reagents stained the Lipid bodies. However, BODIPY-FL-C_{12} is a saturated FA analog which specifically label the fatty acid present within the lipid bodies. Therefore, transfer of BODIPY-FL-C_{12} from Lipid bodies to the target organelles is used to determine the trafficking of fatty acids in various studies by pulse-chase experiments [Developmental Cell (2015) 32, 678–692; Cell Metabolism (2018) 27, 886–897]. Therefore, we have pre-labeled the FAs present in LB in macrophages with BODIPY-FL-C_{12} (FLC_{12}) and then the cells are infected with *Leishmania*. Subsequently, the transport of labelled FAs from LBs to Ld-PVs is determined by detecting the BODIPY-FL-C_{12} on or within the LAMP1 stained Ld-PVs. Our results have shown that Ld-PVs acquire FLC_{12}-labelled FAs from LBs. Thus, we have used a well-established method to determine the transport of fatty acids from LB to Ld-PV.

Minor issues:

Question 1: Often it is not clear whether the authors talk about host or parasite molecules (gp63, Rab18, TRAPPC9,…).

Response 1: We have appropriately mentioned *Leishmania* molecules and macrophage molecules in the revised manuscript as suggested by the reviewer.

Question 2: If the degradation of miRNA is indeed via dicer, how come that only 40 miRNA are affected – one would expect a more global impact? (The authors themselves ask the questions why there are also miRNAs that are upregulated.).

Response 2: I would like to inform that we have discussed this issue in the discussion. We have stated that *Leishmania* infection downregulates expressions of 40 miRNAs and also upregulates the expressions of about 55 miRNAs in infected macrophages which are possibly not Dicer dependent. I would like to submit that recently multiple non-canonical miRNA biogenesis pathways have been identified which are grouped into Drosha/DGCR8-independent and Dicer-independent pathways [Nature (2010) 465, 584–589; Proc Natl Acad Sci U S A (2016) 113, E1881-9; Nucl Acids Res. (2017) 45, 10369–10379; Front. Endocrinol. (2018) 9,402]. Therefore, we have stated that "55 miRNAs in infected macrophages which are possibly not Dicer dependent". However, in the present manuscript we have shown that Ldgp63 degrade the dicer and thereby inhibits the expression of miR-1914-3p. However, based on the fact that miRNA biogenesis is also regulated by multiple non-canonical pathways, we have stated in the revised manuscript as 'upregulated miRNA may be processed by non-canonical pathway' and cited the above references in the revised manuscript.

Question 3: Discussion: A figure outlining the proposed mechanism would be useful.

Response 3: We have provided model of the proposed mechanism as last figure of the reviewed manuscript which reviewer has missed.

Question 4: The text states "Leishmania induced more than 2.5-folds expression of Rab18", whereas neither quantification in Fig. 1a or 1b shows this.

Response 4: I would like to submit that our data showed about 2.5-folds expression of Rab18 in infected macrophages. However, to avoid controversy, we have stated 'more than two folds' in the revised manuscript.

Question 5: The manuscript needs to be checked for grammar - in particular the use of articles, plural forms and the use of "like/etc.".

Response 5: We are sorry for the grammar and the use of articles error. We have carefully edited the revised manuscript.

Question 6: The title, abstract, introduction and discussion imply that this study is relevant to all Leishmania species, however, technicallly only Leishmania donovani was investigated.

Response 6: We have stated *Leishmania donovani* in the revised manuscript as suggested by the reviewer.

Question 7: Introduction of abbreviations e.g. GEF

Response 7: We have included the full form of GEF in the abbreviation list in the revised manuscript.

Reviewer 2:

Comment: Sood and colleagues investigated how Leishmania modulates the host cell traffic to get the lipid bodies in the parasitophorous vacuole. First, they observed the increased expression of Rab18 and its GEF, TRAPPC9, in L. donovani-infected macrophages. Rab18 is a well-known small GTPase involved in intracellular traffic, especially of lipid bodies. As

miRNAs are imputed as major post-transcriptional regulators, they compared their expression in infected and uninfected cells. Among the reduced miRNAs was mi-1914-3p, which was found to be complementary to Rab18 and GEF RNAs domains. This regulation loop was well demonstrated in the MS. Following, they showed the increase of biogenesis and recruitment of lipid bodies by the parasitophorous vacuole. The authors also showed that the parasite metalloprotease GP63, when transfected in HeLa cells, reduced the expression of Dicer. The final finding of the MS is the demonstration that modulating the levels of mi-1914-3p or Rab18 directly interferes with fatty acids colocalization with parasitophorous vacuoles. They correlate the lower availability of fatty acids with the minor development of amastigotes (what they call parasite death), although they do not establish a relationship between these two events. The MS is well-designed, has a good rationale, and addresses an exciting cell biology question.

Response: We are happy to know that reviewer has nicely summarized our results and commented that "finding of the manuscript is the demonstration that modulating the levels of mi-1914-3p or Rab18 directly interferes with fatty acids colocalization with parasitophorous vacuoles". He has also found our "manuscript is well-designed, has a good rationale, and addresses an exciting cell biology question".

Major issues:

Question 1: Although I do not think it is necessary to conduct additional experiments, the presented data do not support some conclusions, so they must be reformulated, and I considered them major issues.

Reply 1: We are happy to know that the reviewer has recommended that no additional experiments are required in the present manuscript. However, he has suggested reformulating some of the conclusions in the manuscript which we have provided in the revised manuscript.

Question 2: The title ("Leishmania highjack host lipid body to acquire fatty acids…") and the justificative in the introduction point the fatty acids acquisition as the main question to be addressed in the manuscript, but, actually, what is shown is the colocalization of lipid bodies and parasitophorous vacuoles. No acquisition of fatty acids by parasites is evaluated. Although fatty acids are significant constituents of lipid bodies, other molecules, such as cholesterol, phospholipids, and even some proteins, are present. I suggest reformulating the title.

Reply 2: I would like to submit that we have used BODIPY™ 493/503 and LipidTOX™ to stain the LBs and anti-perilipin antibody is used to detect lipid droplet-associated protein. Thus, these reagents stained the Lipid bodies and used for the recruitment of LBs by LdPV as agreed by the reviewer. However, BODIPY-FL-C_{12} is a saturated FA analog that specifically labels the fatty acid present within the lipid bodies. Therefore, transfer of BODIPY-FL-C_{12} from Lipid bodies to the target organelles is used to determine the trafficking of fatty acids in various studies by pulse-chase experiments [Developmental Cell (2015) 32, 678–692; Cell Metabolism (2018) 27, 886–897]. We have used the same well-established method to determine fatty acid acquisition by LdPV using FLC_{12}-labelled FAs in LBs by pulse-chase experiments. I think title is appropriate as we have shown the fatty acid recruitment by LdPV. Moreover, we have added a new figure in the revised manuscript showing that the addition of fatty acids supplement reversed the inhibition of parasite growth in mi-1914-3p treated macrophages indicating that parasites acquire fatty acids by enhanced expression of Rab 18

via the downregulation of mi-1914-3p in infected macrophages. Therefore, we believe that title is appropriate. Moreover, this title is also agreed by other reviewers.

Question 3: In the first sentence of the author summary, it is stated that "Leishmania donovani is unable to synthesize fatty acids and depends on its host to scavenge it for its survival". The fatty acid synthesis in Leishmania has already been described. For a recent review, see Arya R, Dhembla C, Makde RD, Sundd M, Kundu S. An overview of the fatty acid biosynthesis in the protozoan parasite Leishmania and its relevance as a drug target against leishmaniasis. Mol Biochem Parasitol. 2021 Nov;246:111416. doi: 10.1016/j.molbiopara.2021.111416. Epub 2021 Sep 21. PMID: 34555376. Please, revise this throughout the entire text.

Reply 3: I would like to submit that fatty acid biosynthesis pathway in not fully characterized in *Leishmania* and parasite salvage fatty acids from host cells [Nat Rev Microbiol. (2017) 5, 287]. The fatty acid biosynthesis pathway highlighted in the referred paper is based on the similar molecule identified in *Trypanosoma*. Even some molecules of this pathway present in *Trypanosoma* are absent in *Leishmania* [Mole Biochem Parasitol (2014) 196, 61–70]. Since *Leishmania* belongs to the same trypanosomatid family, it was predicted that this parasite may also possess the ability of de novo biosynthesis of fatty acids. However, *in silico* analyses have shown that *Leishmania* encode for type II fatty acid synthase (FASII) as well as the fatty acid elongase (FAE) but their role in fatty acid biosynthesis in *Leishmania* is not yet characterized [J of Biomed Biotech (2009) 2009, 1-8]. Thus, *Leishmania* primarily obtain required fatty acids from host cells. Subsequently, internalized FAs are activated in parasite to CoA thioesters by acyl-CoA synthetases (ACS). This acylation represents a vectorial transport of FAs which are then available for subsequent metabolism [Mol Biochem Parasitol (1980)1,177–86]. However, the structure of the acyl carrier protein of *Leishmania major* are found to be different in their transferase binding domain [Biochemistry (2015) 54, 5632–5645]. Moreover, it has been shown that free fatty acids and cholesterol are increased in amastigotes in host cells, whereas triglycerides and ergosterol are decreased. Regarding fatty acid composition, a significant increase of n-7 fatty acids are observed in amastigotes whereas total n-6 fatty acids are decreased in PL [Lipids (2017) 52,433–441]. These studies demonstrates that *Leishmania* acquire fatty acids from their host cells as the parasite possibly does not have complete fatty acid biosynthetic pathway or it is not fully characterized yet. We have modified the statement in the revised manuscript as "Leishmania donovani scavenge the fatty acids from the host cells for its survival but the mechanism is not clearly known" instead of "Leishmania donovani is unable to synthesize fatty acids and depends on its host to scavenge it for its survival".

Question 4: Please, see these detached sentences from the manuscript:
- Abstract: "Moreover, overexpression of miR-1914-3p in macrophages significantly inhibits the recruitment of LBs and thereby suppresses the growth of parasites in macrophages as parasites are unable to acquire fatty acids."
- Author summary: "We have also shown that overexpression of miR-1914-3p in macrophages significantly inhibits the recruitment of LBs and thereby suppresses the growth of parasites in macrophages as parasites are unable to acquire fatty acids."
- Results: "To determine whether Rab18 function is necessary for acquiring fatty acids from LBs in the survival of Leishmania in macrophages…"
"These results indicated that parasite acquires fatty acid from LBs for its survival."
- Discussion: "These results demonstrate that overexpression of Rab18 by downregulating the

expression of miR-1914-3p by Leishmania is essential to acquire FAs from host LBs for the survival of parasites in macrophages."

Reply 4: We have modified the text appropriately in the revised manuscript as suggested by the reviewer.

Question 5: The experimental approach was to modulate the levels of mi-1914-3p or Rab18 and to evaluate the localization of fatty acids derived from lipid bodies and the number of amastigotes in each case. So, several points are raised from the above sentences: - The parasite viability was not accessed, just its proliferation over time. Then, it sounds too strong mentioning parasite survival.

Reply 5: I would like to submit that if parasite does not proliferate in macrophages which means that parasites in not surviving in host cells. However, we have used proliferation/multiplication of the parasites in the revised manuscript as indicated by the reviewer.

Question 6: The fatty acid incorporation by the parasite was not addressed at any time in the manuscript, just its availability from the lipid bodies (other possible pathways, such as LDL uptake, were not evaluated) in the parasitophorous vacuole. So, again sounds too strong the statement "thereby suppresses the growth of parasites in macrophages as parasites are unable to acquire fatty acids".

Reply 6: I am sorry to state that the reviewer has misinterpreted our data with BODIPY-FL-C_{12} which specifically labelled the fatty acids. We have used a well-established method to determine transfer of fatty acids using FLC_{12}-labelled FAs [Developmental Cell (2015) 32, 678–692; Cell Metabolism (2018) 27, 886–897].

Question 7: miR1914-3p and Rab18 are involved in other cell processes. Then, it is not possible to state that parasite growth is impaired due to the lack of fatty acids from the lipid bodies. No reversion experiments were conducted, such as fatty acid supplementation.

Reply 7: I do agree with the reviewer that miR1914-3p and Rab18 may be involved in other cellular processes. However, we have shown that *Leishmania* by its gp63 degrades dicer and down regulated miR1914-3p expression in host cells and thereby overproduces Rab18. Subsequently, Rab18 drives LB biogenesis and then LB are recruited by LdPV to acquire fatty acids. Overexpression of the miR1914-3p in macrophages blocks the LB recruitment and thereby inhibits parasite multiplication suggesting that parasite growth is impaired due to the lack of fatty acids under this condition. I agree with the reviewer that fatty acid supplementation could prove this point more clearly. Accordingly, we have added a new figure in the revised manuscript as shown below showing that the addition of fatty acids supplement reversed the inhibition of parasite growth in mi-1914-3p treated macrophages indicating that parasites acquire fatty acids by enhanced expression of Rab 18 via the downregulation of mi-1914-3p in infected macrophages. The inclusion of the fatty acid supplementation data in the revised manuscript certainly strengthens our observation as suggested by the reviewer.

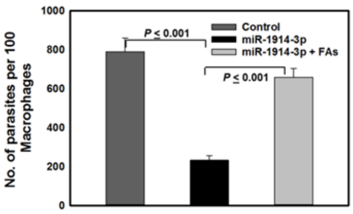

Question 8: Page 6 – "Consequently, we showed that gp63, a metalloprotease in Leishmania, degraded the Dicer in HeLa cells". The data presented are related to the reduced dicer expression in the transfected cells. It was not shown whether gp63 is able to degrade dicer directly, nor how this enzyme would be in contact with dicer inside the cell.
Page 6 – "These results demonstrated that Leishmania degrades the Dicer by its gp63 and thereby reduces the expression of miR-1914-3p…". The only presented experiment involving gp63 was transfecting HeLa cells with the metalloprotease and evaluating the dicer expression. No relationship among gp63, dicer degradation, and expression of miR-1914-3p was stablished.

Reply 8: We have revised the text in light of the reviewer suggestions. Reviewer has stated that "No relationship among gp63, dicer degradation, and expression of miR-1914-3p was established". However, we have shown in the Fig. 3f of the reviewed manuscript that the expression of gp63 alone reduces the expression of dicer. Moreover, I am sorry to inform that this is in sharp contrast with Reviewer 1 statement where he has said that "Through a set of experiments authors have shown that dicer is degraded by gp63, which reduces the expression of miR-1914-3p. This in turn upregulates Rab18 and TRAPPC9, which induces the recruitment of fatty acid-containing lipid bodies to the parasitophorous vacuole. Accordingly, reviewer 1 has commented that "This set of events provide a significant, comprehensive insight into the mechanism of fatty acid uptake by *Leishmania donovani*". The same is also agreed by the Reviewer 3.

Minor issues:

Question: Page 10: "Leishmania do not have complete lipid synthesis pathway and therefore, parasite needs to scavenge lipids from the host cells [43]." This sentence is vague (what would be a complete lipid synthesis?), and the reference is misused. This reference does not address this question. This sentence repeats below in text.

Response: We have appropriately modified the text in the revised manuscript as stated in our previous reply.

Reviewer 3:

Comment: In this fascinating new manuscript, the focus on the connection between lipid bodies (or lipid droplets) and the PV of Leishmania Donovani. The authors use gene expression analysis, confocal microscope and manipulation of expression of Rab18 and miR-1914-3p to conclude that Leishmania manipulated Dicer2 levels, which in turn control miR-1914-3p levels, increase Rab18 and TRACC9 to deliver LBs to the PV.

Response: We appreciate the reviewer for finding our manuscript fascinating.

Major Issues:

Question 1: Most the imaging does NOT unequivocally identify the PV, with the exception of Figure 5 that includes LAMP1 staining that outlines the PVs. In all the other figures, it is not possible to accurately identify the PV and determine if the conclusions about association of PV with LB and/or Rab18 are accurate. This needs to be thorough addressed with. new data. It would also be an significant improvement if the Leishmania parasite were visualized by something more specific then small little dotes of PI or Draq5 stain.

Reply 1: We have included new data showing the PV staining by LAMP1 in almost all figures in the revised manuscript as suggested by the reviewer. I would like to submit that we have marked the PV with dotted lines based on the size of the PV (10 µm) as demonstrated earlier in the reviewed manuscript. I would like to inform that staining of the parasite nucleus is the only way to visualized parasites in infected cells and this method is well established and used by all investigators in the field.

Question 2: The authors claim to "unequivocally demonstrate" their model, however (even ignoring the issue above) the data as a whole are consistent with the conclusion but do not prove/show/demonstrate the model. The authors need to find a way to interfere with their proposed pathway: GP63-|Dicer-|miR-1914-3p-|Rab18 and LB recruitment to the PV. Artificially over-expressing miR-1914-3p nor knocking down Rab18 are not the same as blocking this pathway; these experiments do no provide unequivocal demonstration. Moreover, Figure 5 that aims to demonstrate fatty acid recruitment to the PV is not convincing. Seems like there is more FA signal in the general vicinity of the PVs, as seen in the image of the whole cells, but it is not clear from the inset/zoom in that the actual fusion or delivery of the FA to the PV membrane, and certainly not into the PV, even at 24hr has occurred. Also, the time course shown in Figure 5A is critically important for the conclusion of this paper, but only 1 cells is shown for each time point and there is no quantitation.

Reply 2: We have withdrawn the word "unequivocally demonstrate" in the revised manuscript. I would like to submit that we have provided mechanistic details of the proposed pathway: GP63-|Dicer-|miR-1914-3p-|Rab18 and LB recruitment to the PV in the reviewed manuscript as follows: i. Ldgp63 degrade the dicer; ii. *Leishmania* infection reduces the expression of miR-1914-3p in infected cells and thereby enhances the expression of Rab18; iii. Rab18 induces the lipid body biogenesis and recruit LB and Rab18 on PV; iv. Fatty acids in LB labelled with FLC_{12} transferred from LB to LdPV; v. recruitment of LB is blocked by the overexpression of miR-1914-3p. Thus, we have shown that *Leishmania* reduces the expression of miR-1914-3p to acquire fatty acids from LB and this process is blocked by the overexpression of miR-1914-3p. We are not claiming the actual fusion of LB with LdPV. Here we have shown that transfer of fatty acid to LdPV membrane and inside PV. I hope reviewer will agree that it is not expected to see all FA signal transferred to PV and there should be other FA signal in LB in the general in the cells. We have observed several cells and provided one representative image in the reviewed manuscript. We have provided quantitation in the revised manuscript.

Question 3: Another important issue, but more about language, is that the authors often write that Rab18 or TRACC is "overexpressed" in the context of *Leishmania* infection. This has the connotation of experimental over expression, for example with transfection of an expression plasmid. However, in fact, this is a mild (about 2x fold according to the data presented) increase in protein and mRNA expression observed with Leishmania infection. Different language should be used to make this point more clearly. Moreover, it raises a question: is

this 2x fold change in Rab18 (and TRACC9) really sufficient to drive this dramatic change in LB localization?

Reply 3: We have used ‘enhance expression’ in the revised manuscript. I agree with reviewer that we have observed about 2x fold enhanced expression of Rab18 and TRACC9 by mRNA and proteins in *Leishmania* infected cells. I would like to submit that 2x fold enhanced expression of these molecules are sufficient to drive the localization and functions of these proteins as we have shown in the present manuscript as well as in our previous studies.

Minor issues:

Question 1: Almost all the figures are spread across multiple pages. This is not usually acceptable at most journals.

Reply 1: We have submitted all figures across multiple pages as PDF file for initial review process. We have submitted appropriate figure files in the revised manuscript.
