# Supplementary material for: Leishmania highjack host lipid body for its proliferation in macrophages by overexpressing host Rab18 and TRAPPC9 by downregulating miR-1914-3p expression
Source: PLoS Pathog. 2024 Feb 27;20(2):e1012024. doi: 10.1371/journal.ppat.1012024 (PMC10898768; doi:10.1371/journal.ppat.1012024)
Supplement: Attachment — Submitted filename: Point by Point response.pdf [file ppat.1012024.s007.pdf]

## Point by Point response:

**Reviewer 1:**

**Comment:** No comment on the revised manuscript.

**Reviewer 2:**

**Question 2**:

**Reviewer reply:** The authors assume that the mere access of fatty acids to the PV automatically results in their incorporation into the parasites, which cannot be true. They did not demonstrate through any technique that the labeled fatty acid is inside the amastigotes. Furthermore, the fact that BODIPY-FL-C12 has been found inside the PV does not rule out the involvement of other constituents of LBs. In other words, the presented data show that fatty acids from LBs are in the PV, but this does not necessarily mean that they are incorporated by the parasites, nor does it imply that they are the only significant molecules from LBs relevant to the observed phenomenon in the sequence. Additionally, the fact that the other reviewers have not mentioned the title in their reviews does not necessarily mean that they agree.

**Our Response:** I would like to appreciate the reviewer for agreeing that BODIPY-FL-C12 which specifically labels the fatty acid is transferred from LBs to LdPV. Moreover, we have shown in the revised manuscript that fatty acids supplement reverses the inhibition of parasite growth in miR-1914-3p treated macrophages. Thus, it appears that parasites acquire fatty acids from LB in macrophages for their intracellular proliferation. Moreover, it has been shown that single amastigote of *L. donovani* resides in individual PV tightly wrapped by the PV membrane [Cellular Microbiology (2010) 12, 1480–1494; J. Parasitol (2006) 92, 1162-1170; Annals of Tropical Medicine and Parasitology (1974) 68, 167-176; J. Protozoology (1964) 11, 166-191]. Thus, the incorporation of fatty acids within LdPV possibly suggests the acquisition of fatty acids in the parasite. I would like to submit that due to technical limitations, it is difficult to show the incorporation of fatty acids in parasites inside the macrophages. We have also seen that LAMP1 labelled PV is in close association with GFP overexpressed parasites in macrophages. However, we are unable to use GFP overexpressed parasites as fluorescence is badly quenched in low pH of Ld-PV. Another technical limitation is that BODIPY-FL-C12 is green and LAMP1 is red. I do agree with the reviewer that lipid bodies contain other molecules, such as cholesterol, phospholipids, and even some proteins. We have only shown the requirement of fatty acids from LB by LdPV in macrophages for the growth of the parasites. We do not know role of other molecules present in LB for the proliferation of parasites in macrophages. We have modified the re-revised manuscript accordingly and changed the title of manuscript as "Leishmania highjack host lipid body for its proliferation in macrophages by overexpressing host Rab18 and TRAPPC9 by downregulating miR-1914-3p expression" as suggested by the reviewer.

**Question 5:**

**Reviewer reply:** Thank you for adjusting the text.

**Our response:** I also thank the reviewer for accepting the text in the revised manuscript.

**Question 6:**

**Reviewer reply:** I regret to note that the authors may not fully grasp that the colocalization of a molecule in the PV does not constitute proof that the molecule is inside the parasite. Furthermore, this does not rule out the possibility of the involvement of other LB constituents, nor does it exclude other potential sources of fatty acids, as mentioned.

**Our response:** I do agree with the reviewer that the colocalization of labelled fatty acids from LB in the PV does not necessarily prove that the molecule is inside the parasite. However, a single amastigote resides in individual PV, and fatty acids supplementation reversed the inhibition of parasite growth in miR-1914-3p treated macrophages suggest that parasites acquire fatty acids from LB. I have modified our statement in the re-revised manuscript as “Our results suggest that parasites acquire fatty acids from LB” as suggested by the reviewer.

**Question 8:**

**Reviewer reply:** I would like the authors to specify where they demonstrate the degradation of Dicer by GP63. I was unable to find the Western blot illustrating the degradation of Dicer, as well as an explanation of how a major surface glycoprotein can gain access to Dicer within the parasite.

**Our response:** I would like to state that we have shown in the Fig. 3e, THP-1 differentiated macrophages were infected with *L. donovani* significantly reduces the expression level of Dicer in infected cells. Subsequently, in Fig.3f we showed that the expression of Dicer is also significantly reduced in HeLa cells transfected with Ldgp63. Accordingly, we have stated in the results section of the revised manuscript that “We found that *Leishmania* infection reduces the level of Dicer in infected macrophages (Fig. 3e). Consequently, our results showed that Ldgp63, a metalloprotease in *Leishmania*, also reduces the Dicer expression in HeLa cells (Fig.3f)”. It has been shown that Ldgp63 secreted by *Leishmania* hijacks host macrophage signaling by degrading several critical serine/threonine kinases and several transcription factors to alter gene expression in macrophages [Microbes Infect. (2012)14, 1377-1389]. Therefore, reduced expression of host cell Dicer in infected macrophages is possibly due to the degradation by Ldgp63. I would like to submit that we have stated in the revised manuscript that *Leishmania* infection reduces the expression of dicer in macrophages by their metalloprotease Ldgp63. I am sorry that Dicer degradation was mentioned in one place in the revised manuscript which also we have changed in the re-revised manuscript. I would also like to clarify that we are referring to the expression of host Dicer in infected macrophages rather than Dicer within the parasite.

**Minor issues:**

Reviewer #2: (No Response)

**Reviewer 3:**

**Comment:** The first round of critiques have been thoroughly addressed. Major issues are addressed with new data, especially LAMP1 staining throughout and also the FA

supplementation experiment. In addition, the language has been adjusted so that conclusions and interpretations are not overstated.

**Our response:** I appreciate the reviewer for finding our revised manuscript thoroughly addressed all concerns of the reviewer.

**Comment:** One improvement that should be easy, but would be a major improvement: in figure 5A, can the "acquired" FA be noted with an arrow or similar...what are the authors scoring as "acquired". This was a key issue in the prior reviews and could be clarified further, although the data is all there, I believe.

**Our response:** We have incorporated an arrow for the FA in the Fig. 5a in the revised manuscript as suggested by the reviewer. FA (green) acquired by LAMP1 stained LdPV (red) was determined by counting the presence of green dots (FA) in/on within the LdPV of about 100 LdPV in infected macrophages using ImageJ software and results are expressed as percentage of LdPV acquiring FA.

**Minor issues:**

**Comments:** Be sure all graphs are completely labeled. For example, what are the different sets of bars in 4d? The x-axis is not labeled.
I think there is a stray Asterix and P value noted on the lower half of 4e (the main text says no difference).
5c graph should have x-axi labels too...presumably different time points.

**Our response:** We are sorry for the mistake. We have appropriately corrected all figures in the re-revised manuscript as suggested by the reviewer.
